# Association between homocysteinemia and mortality in CKD: A propensity-score matched analysis using NHANES-National Death Index

**DOI:** 10.1097/MD.0000000000030334

**Published:** 2022-09-09

**Authors:** Je Hun Song, Hyuk Huh, Eunjin Bae, Jeonghwan Lee, Jung Pyo Lee, Jong Soo Lee, Gwang Sil Kim, Kyung Don Yoo

**Affiliations:** a Department of Internal Medicine, Ulsan University Hospital, University of Ulsan College of Medicine, Ulsan, South Korea; b Department of Internal Medicine, Pusan Paik Hospital, Inje University College of Medicine, Busan, South Korea; c Department of Internal Medicine, Gyeongsang National University Changwon Hospital, Changwon, South Korea; d Department of Internal Medicine, Seoul National University Boramae Medical Center, Seoul, South Korea; e Department of Internal Medicine, Seoul National University College of Medicine, Seoul, South Korea; f Department of Internal Medicine, Sanggye Paik Hospital, Inje University College of Medicine, Seoul, South Korea.

**Keywords:** cardiovascular disease, chronic kidney disease, homocysteine, mortality

## Abstract

Hyperhomocysteinemia (HHcy) is considered a risk factor for cardiovascular disease (CVD), including chronic kidney disease (CKD). In this study, we investigated the association between levels of serum homocysteine (Hcy) and mortality, inferred from the presence of CKD. Our study included data of 9895 participants from the 1999 to 2016 National Health and Nutrition Examination Surveys (NHANES). Multivariable-adjusted Cox proportional hazard models using propensity-score, were used to examine dose-response associations between Hcy level and mortality. A total of 9895 participants, 1025 (10.3%) participants were diagnosed with CKD. In a multivariate Cox regression analysis including all participants, Hcy level was significantly associated with all-cause mortality in the nonCKD group, compared to the 1^st^ quartile in the fully adjusted model (2^nd^ quartile: hazard ratio (HR) 1.75, 95% confidence interval (CI) 1.348–2.274, *P* < .001; 3^rd^ quartile: HR 2.22, 95% CI 1.726–2.855, *P* < .001; 4^th^ quartile: HR 3.77, 95% CI 2.952–4.830, *P* < .001). However, this finding was not observed in the CKD group. The observed pattern was similar after propensity score matching. In the nonCKD group, overall mortality increased in proportion to Hcy concentration (2^nd^ quartile: HR 2.19, 95% CI 1.299–3.709, *P *= .003; 3^rd^ quartile: HR 2.60, 95% CI 1.570–4.332, *P* < .001; 4^th^ quartile: HR 3.72, 95% CI 2.254–6.139, *P* < .001). However, the risk of all-cause mortality according to the quartile of Hcy level, did not increase in the CKD group. This study found a correlation between the Hcy level and mortality rate only in the nonCKD group. These altered risk factor patterns may be attributed to protein-energy wasting or chronic inflammation status, that is accompanied by CKD.

## 1. Introduction

The prevalence of chronic kidney disease (CKD) increases with age, along with the risk of cardiovascular disease (CVD).^[1,2]^ Prior evidence suggest an association between CKD and CVD through various shared mechanisms. Moreover, recent research presented the epidemiologic and biologic mechanism associating CVD and CKD, not only among patients with advanced CKD undergoing dialysis, but also those with mild to moderate CKD.^[3,4]^ Homocysteine may have a role in the development of CVD in patients with mild CKD, notwithstanding the paucity of research on this topic. Previous research has investigated homocysteine, as a potential risk factor for CVD and CKD in the general population.^[5]^ Based on “Reverse epidemiology”, where traditional CVD/mortality risk factors appear protective in advanced CKD, we would also clarify whether this has been observed for Hyperhomocysteinemia (HHcy), or just for traditional risk factors like obesity. In particular, reverse epidemiology was observed in the mortality of advanced CKD patients, compared to that of the general population group.^[6]^ Further, this effect was highly associated with the malnutrition-chronic inflammation hypothesis for obesity, using body mass index (BMI) and dyslipidemia in CKD patients.^[7–9]^

Notably, dialysis patients were associated with low homocysteine (Hcy) and mortality.^[10,11]^ A randomized HOST-trial in 2007 announced vitamin combination therapy for 2056 CKD and end-stage renal disease (ESRD) patients.^[12]^ In this trial, while the level of Hcy significantly decreased, it did not improve the CVD outcome. Moreover, as prior research has focused on traditional risk factors such as high blood pressure and Diabetes Mellitus (DM), there is limited research on the factors influencing increased CVD risk among CKD patients. Therefore, the purpose of this study was to systematically identify CKD patients using national open-source data, along with the identification of change in mortality according to the Hcy level of CKD and nonCKD patients, before and after propensity score matching. This gave the results of this study a higher scope of application in future research.

## 2. Materials and Methods

### 2.1. Study population and ethics statement

**Figure 1.**
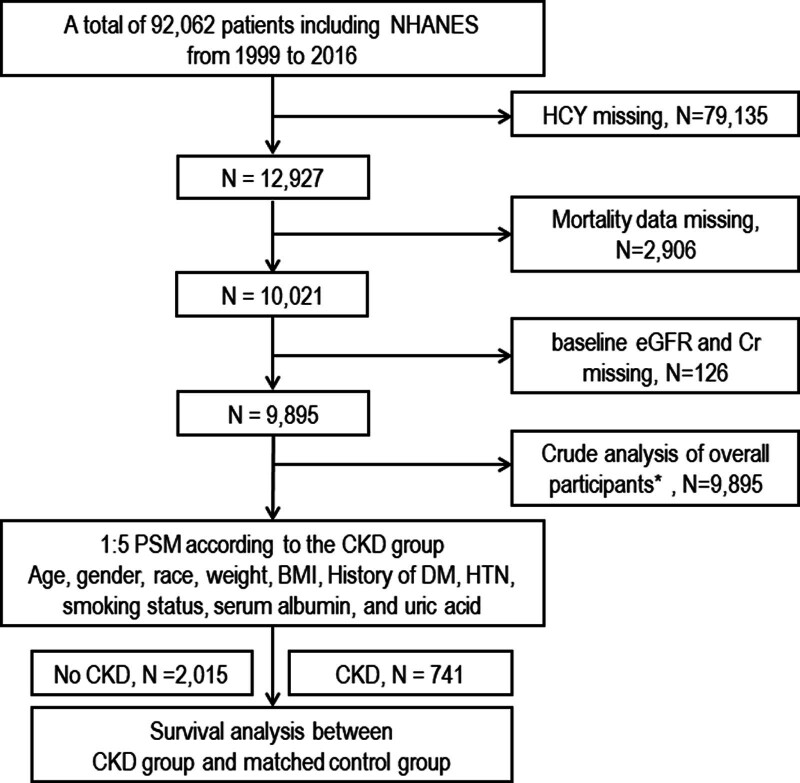
Study flow chart.

The National Health and Nutrition Examination Surveys (NHANES) is a large cross-sectional survey conducted for citizens of the United States, providing basic and clinical information on the prevalence and risk factors of chronic diseases. We used anonymized information of participants included in the NHANES from 1996 to 2016. This study protocol received approval from the National Center for Health Statistics (NCHS) Institutional Review Board of NHANES.^[13]^ We included 9895 out of 92,062 participants, by excluding those with missing estimated Glomerular Filtration Rate (eGFR), Hcy level, and mortality data (Fig. 1). The NCHS Research Ethics Review Board (ERB) approved the NHANES protocols (protocol #98-12, #2005-06 and #2011-17, available site: https://www.cdc.gov/nchs/nhanes/irba98.htm), and written informed consent was obtained from NHANES participants, with all procedures approved by the NCHS Research Ethics Review Board. This research has been carried out in accordance with the Declaration of Helsinki. The NHANES performance of adult specimens was evaluated based on a method described in a previous study.^[14]^

### 2.2. Measurement of homocysteine and covariates

Blood samples from NHANES participants are stored, processed, and analyzed according to standardized protocols, for measurement of total plasma Hcy of participants. The Abbott Homocysteine assay was used to measure Hcy in plasma. This laboratory method is a fully automated fluorescence polarization immunoassay from Abbott Diagnostics. Further, total plasma Hcy concentrations were calculated using a machine-stored calibration curve. Other laboratory data such as serum creatinine, albumin, and uric acid were also analyzed and self-reports were used to collect information on demographic variables of participants including age, gender, race, education level, and smoking status.

### 2.3. Definitions of variables and outcomes

CKD was defined as an eGFR < 60 mL/min/1.73 m^2^. Hypertension was defined as systolic blood pressure (SBP) ≥ 140 mm Hg and/or a diastolic blood pressure (DBP) ≥ 90 mm Hg. DM was defined as hemoglobin A1c ≥ 6.5%, self-reports of prior diagnosis of DM, or consumption of medications for DM. The primary outcome of the study was all-cause mortality, which was defined using the National Death Index (NDI) mortality data.

### 2.3. Statistical analysis

We divided study population into 2 groups (i.e., CKD and nonCKD group), based on the value of eGFR. For group comparisons, t-test and χ2 test were used for continuous variables and proportions, respectively. Cox regression models were applied to calculate the hazard ratios (HRs). Further, 95% confidence intervals (CIs) were set for homocysteine and all-cause mortality. In Model 3, we included covariates that had a significant association amongst themselves (e.g., age, gender, serum albumin, urine albumin creatinine ratio (UACR), smoking status, BMI, concurrent history of hypertension, and DM; *P* < .05). We used Kaplan–Meier survival curves to calculate all-cause mortality. Supplemental Digital Content (Figure S1, http://links.lww.com/MD/H158) shows the distribution of standardized difference between the groups, after the propensity-score matching. The propensity score and standardized differences were used to compare baseline characteristics between the 2 groups. The former was used to balance covariates between the 2 groups, for reduction of bias effects of between-group differences in baseline characteristics, on measured effects of the CKD. The nearest neighbor matching method and 1:5 matching algorithm without replacement, were used to select a match in the control group, for each individual in the CKD group (see Supplemental Digital Content (Figure S1, http://links.lww.com/MD/H158), which demonstrates standardized differences pre and post matching, change the difference of group variation).

## 3. Results

### 3.1. Baseline characteristics of the study population

The average age of the study participants was 47 (± 20) years, with a sample of 48.4% males. Among the study population, 3771 (38.1/%) had hypertension, whereas 1117 (11.3%) had DM. The CKD group was older in age (CKD: 73 ± 11 years; nonCKD: 44 ± 18 years, *P* < .001) and had a lower level of education, compared to the nonCKD group. Moreover, in the CKD group, BMI, SBP, DBP, uric acid and the prevalence of hypertension and DM were significantly high, whereas serum albumin level was low (Table 1).

**Table 1:** Baseline characteristics of the study population according CKD.

Variables	Total (N = 9895)	CKD (N = 1025)	nonCKD (N = 8870)	*P*
Age (years)	47 ± 20	73 ± 11	44 ± 18	<0.001
Men, %	4789 (48.4)	487 (47.5)	4302 (48.5)	0.553
Race/ethnicity, %				<0.001
nonHispanic white	5005 (50.6)	640 (62.4)	4365 (49.2)	
nonHispanic black	2145 (21.7)	253 (24.7)	1892 (21.3)	
Other-Hispanic	309 (3.1)	14 (1.4)	295 (3.3)	
Mexican-American	2033 (20.5)	86 (8.4)	1947 (22.0)	
Other	403 (4.1)	32 (3.1)	371 (4.2)	
Education level, %				<0.001
<12 years	2615 (26.4)	378 (36.9)	2237 (25.2)	
12–15 years	2277 (23.0)	272 (26.5)	2005 (22.6)	
≥16 years (College)	2615 (26.4)	212 (20.7)	2403 (27.1)	
≥16 years (Graduate)	1809 (18.3)	157 (15.3)	1652 (18.6)	
Missing (Refuse to answering, or missing)	579 (5.9)	6 (0.6)	573 (6.5)	
BMI (kg/m^2^)	28.5 ± 6.5	29.0 ± 6.2	28.4 ± 6.6	0.012
SBP (mm Hg)	125 ± 21	142 ± 26	123 ± 19	<0.001
DBP (mm Hg)	69 ± 14	66 ± 19	70 ± 13	<0.001
Smoking status, %				<0.001
Never	4783 (51.3)	503 (49.1)	4280 (51.5)	
Ex-smoker	2457 (26.3)	408 (39.8)	2049 (24.7)	
Current	2081 (22.3)	112 (10.9)	1969 (23.7)	
Albumin (g/dL)	4.2 ± 0.4	4.0 ± 0.4	4.2 ± 0.4	<0.001
Uric acid (mg/dL)	5.4 ± 1.4	6.5 ± 1.6	5.2 ± 1.4	<0.001
Estimated GFR (mL/min/1.73m^2^)	92 ± 25	46 ± 12	98 ± 20	<0.001
GFR grade				<0.001
G1	5533 (55.9)	0	5533 (62.4)	
G2	3337 (33.7)	0	3337 (37.6)	
G3a	666 (6.7)	666 (65.6)	0	
G3b	250 (2.5)	250 (24.4)	0	
G4	88 (0.9)	88 (8.6)	0	
G5	21 (0.2)	21 (2.0)	0	
Homocysteine (umol/L)	8.8 ± 4.7	13.5 ± 5.1	8.3 ± 4.3	<0.001
Albumin creatinine ratio (mg/g)	43.1 ± 341.4	215.5 ± 926.5	24.1 ± 175.8	<0.001
				<0.001
A1 (<30 mg/g)	8605 (87.0)	668 (68.9)	7937 (90.6)	
A2 (30–300 mg/g)	946 (9.6)	207 (21.9)	739 (8.4)	
A3 (>300 mg/g)	184 (1.9)	95 (9.8)	89 (1.0)	
Hypertension, %	3771 (38.1)	810 (79.0)	2961 (33.4)	<0.001
Diabetes, %	1117 (11.3)	296 (28.9)	821 (9.3)	<0.001
Crude mortality rate		587 (57.3)	1020 (11.5)	<0.001

CKD was defined as estimated GFR < 60 ml/min/1.73 m^2^,

BMI = body mass index, CKD = chronic kidney disease, DBP = diastolic blood pressure, GFR = glomerular filtration ratio, SBP = systolic blood pressure.

### 3.2. Plasma homocysteine level according to the stage of chronic kidney disease

**Figure 2.**
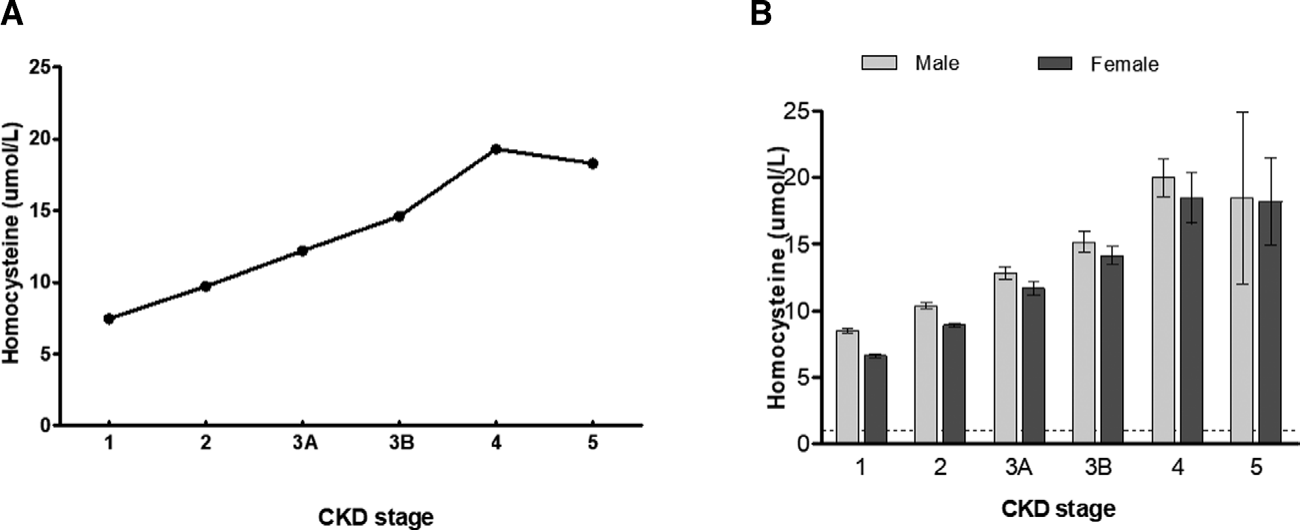
Serum levels of homocysteine according to CKD stage. (A) Levels of Homocysteine according to CKD stage by GFR grade. (B) Levels of Homocysteine according to CKD stage by gender

For total plasma Hcy, the overall mean was 8.8 (± 4.7) umol/L. Participants in the CKD-group had a higher Hcy level, compared to those in the nonCKD group (13.5 ± 5.1 vs 8.3 ± 4.3umol/L, *P* < .001). Although an identical trend from CKD stage 4 to CKD stage 5 was not observed, Hcy levels in the CKD group according to the stages, showed a tendency of gradual increase with the progression from stage 1 to stage 4 (Fig. 2A). The same pattern was observed when analyzed separately by gender (Fig. 2B).

### 3.3. Analysis of all-cause mortality according to per quartile homocysteine level

**Figure 3.**
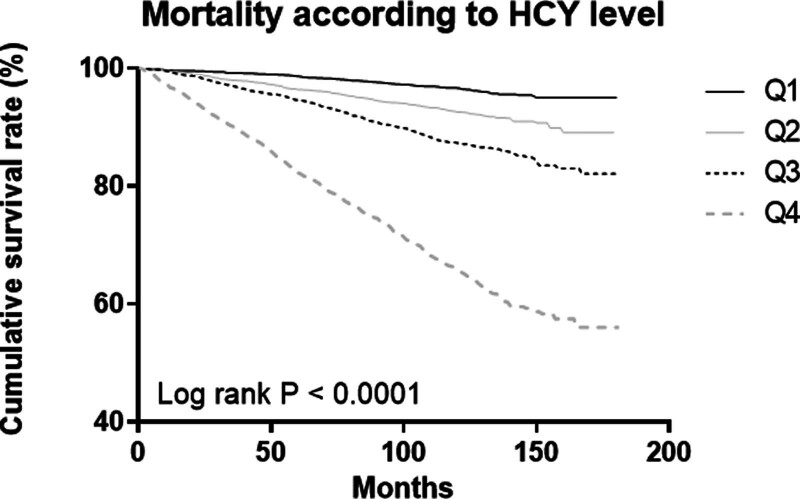
Comparison of the cumulative survival rate between the homocysteine quartile group.

We divided participants into quartiles, based on their Hcy levels. The mortality rate of the overall population increased, with an increase in the level of Hcy (Fig. 3). We used multivariate Cox regression analysis to adjust for age and gender in Model 2; further, we adjusted for age, gender, serum albumin, UACR, smoking status, BMI, concurrent history of hypertension, and DM in Model 3. In a multivariate Cox regression analysis comprising all participants, Hcy level was associated with all-cause mortality, compared to the 1^st^ quartile in Model 3 (2^nd^ quartile: HR 1.75, 95% CI 1.348–2.274, *P* < .001; 3^rd^ quartile: HR 2.22, 95% CI 1.726–2.855, *P* < .001; 4^th^ quartile: HR 3.77, 95% CI 2.952–4.830, *P* < .001). In the nonCKD group, a significant association with all-cause mortality was observed, which was absent in the CKD group (Table 2).

**Table 2:** Hazard ratios of all-cause according to homocysteine level (quartile).

	All (n = 9895)			CKD (n = 1025)			nonCKD (n = 8870)		
	HR	95% CI	*P*	HR	95% CI	*P*	HR	95% CI	*P*
	All-cause mortality								
Model 1*									
Q2	2.07	1.633–2.632	<0.001	0.83	0.293–2.365	0.731	2.07	1.261–2.646	<0.001
Q3	3.60	2.885–4.498	<0.001	1.15	0.467–2.857	0.756	3.17	2.517–4.009	<0.001
Q4	11.36	9.244–13.961	<0.001	2.18	0.905–5.269	0.082	7.60	6.097–9.493	<0.001
Model 2†									
Q2	1.47	1.155–1.872	0.002	0.76	0.270–2.178	0.618	1.43	1.118–1.840	0.005
Q3	1.85	1.474–2.339	<0.001	0.96	0.388–2.379	0.930	1.65	1.298–2.116	<0.001
Q4	3.84	3.073–4.800	<0.001	1.70	0.706–4.127	0.236	2.96	2.328–3.768	<0.001
Model 3‡									
Q2	1.75	1.348–2.274	<0.001	0.88	0.280–2.775	0.830	1.70	1.301–2.240	<0.001
Q3	2.22	1.726–2.855	<0.001	1.23	0.449–3.373	0.687	1.99	1.523–2.604	<0.001
Q4	3.77	2.952–4.830	<0.001	1.93	0.720–5.182	0.191	3.01	2.308–3.941	<0.001

CKD was defined as estimated GFR < 60 ml/min/1.73 m^2^. Reference was Quartile 1 in model 1, 2, 3.

* Model 1: crude.

† Model 2: adjusted for age over 65, gender,

‡ Model 3: adjusted for age over 65, gender, serum albumin, urine albumin creatinine ratio, smoking status, body mass index, concurrent history of hypertension and diabetes mellitus.

CKD = chronic kidney disease, HR = hazard ratio

### 3.4. Analysis pre and post propensity score matching

We performed propensity score matching by dividing the CKD and nonCKD group. Participants were allocated to each group in a 1:5 ratio. We used variables including age, gender, race, BMI, DM, and hypertension, for propensity score matching. Baseline characteristics of the study population after propensity score matching, are presented in Table 3. Post this procedure, we included 2015 and 741 participants in the nonCKD group and CKD group, respectively, after adjusting for variables such as the differences in socioeconomic factors such as race, education level, BMI, and smoking history, serum glucose level were offset between the groups. We observed a decrease in the differences in factors such as the prevalence of DM, hypertension, SBP, serum albumin and uric acid level (Table 3). The risk of mortality according to Hcy level after propensity score matching is presented in Table 4. In the multivariate Cox regression analysis, we found similar results compared to those observed in the crude analysis. The risk of all-cause mortality according to the quartile of Hcy level was not found to increase in the CKD group. However, in the nonCKD group, the Hcy level was associated with all-cause mortality (Table 4).

**Table 3:** Baseline characteristics of the study population according CKD group before and after propensity score matching.

Variables	No CKD (N = 8870)	CKD (N = 1025	*P*	SD	No CKD (N = 2015	CKD (N = 741)	*P*	SD
Age (yr)	44 ± 18	73 ± 11	<0.001	2.439	65 ± 15	71 ± 12	<0.001	0.157
Age over 65 years, %	1405 (15.8)	806 (78.6)	<0.001	1.504	1116 (55.4)	537 (72.5)	<0.001	0.066
Men, %	4302 (48.5)	487 (47.5)	0.553	0.023	1031 (51.2)	367 (49.5)	0.465	0.000
Race/ethnicity, %			<0.001	0.373			0.059	0.000
nonHispanic white	4365 (49.2)	640 (62.4)			1182 (58.7)	460 (62.1)		
nonHispanic black	1892 (21.3)	253 (24.7)			425 (21.1)	173 (23.3)		
Other-Hispanic	295 (3.3)	14 (1.4)			40 (2.0)	12 (1.6)		
Mexican-American	1947 (22.0)	86 (8.4)			275 (13.6)	72 (9.7)		
Other	371 (4.2)	32 (3.1)			93 (4.6)	24 (3.2)		
Education level, %			<0.001				0.116	
<12 years	2005 (22.6)	272 (26.5)			509 (25.3)	202 (27.1)		
12–15 years	2403 (27.1)	212 (20.7)			523 (26.0)	153 (20.6)		
≥16 years	1652 (18.6)	157 (15.3)			350 (17.4)	129 (17.4)		
BMI (kg/m^2^)	28.4 ± 6.6	29.0 ± 6.2	0.012	0.056	29.2 ± 6.4	28.8 ± 6.1	0.116	−0.031
Weight (kg)	80.4 ± 20.7	79.5 ± 19.7	0.191	-0.068	82.0 ± 20.5	79.8 ± 19.7	0.013	−0.036
SBP (mm Hg)	123 ± 19	142 ± 26	<0.001		134 ± 23	141 ± 25	<0.001	
DBP (mm Hg)	70 ± 13	66 ± 19	<0.001		70 ± 15	67 ± 19	<0.001	
Smoking status, %			<0.001	0.156			0.313	−0.012
Never	4280 (51.5)	503 (49.1)			932 (46.3)	351 (47.4)		
Ex-smoker	2049 (24.7)	408 (39.8)			788 (39.1)	296 (39.9)		
Current	1969 (23.7)	112 (10.9)			295 (14.6)	94 (12.7)		
Albumin (g/dL)	4.2 ± 0.4	4.0 ± 0.4	<0.001	−0.301	4.1 ± 0.3	4.1 ± 0.4	0.017	−0.005
Glucose (mg/dL)	97.1 ± 33.4	110.3 ± 41.2	<0.001		107.3 ± 39.9	108.2 ± 41.8	0.608	
Uric acid (mg/dL)	5.2 ± 1.4	6.5 ± 1.6	<0.001	0.798	5.8 ± 1.4	6.1 ± 1.5	<0.001	0.050
Estimated GFR (mL/min/1.73m^2^)	98 ± 20	46 ± 12	<0.001		81 ± 15	48 ± 12	<0.001	
Albumin creatinine ratio (mg/g)	24.1 ± 175.8	215.5 ± 926.5	<0.001		42.3 ± 161.3	221.8 ± 981.0	<0.001	
Diabetes, %	821 (9.3)	296 (28.9)	<0.001	0.422	430 (21.3)	184 (24.8)	0.056	−0.014
Hypertension, %	2961 (33.4)	810 (79.0)	<0.001	1.093	1356 (67.3)	565 (76.2)	<0.001	0.033
Homocysteine (µmol/L)	8.3 ± 4.3	13.5 ± 5.1	<0.001		9.8 ± 4.5	13.1 ± 5.2	<0.001	

Data are expressed as mean (SD). Bold values are statistically significant. CKD was defined as estimated GFR < 60 ml/min/1.73m^2^.

CKD = chronic kidney disease, BMI = body mass index, SBP = systolic blood pressure, DBP = diastolic blood pressure, GFR = glomerular filtration ratio.

**Table 4:** Hazard ratios of all-cause mortality according to homocysteine level (quartile) after propensity score matching.

	All (n = 2756)			CKD (n = 741)			nonCKD (n = 2015)		
	HR	95% CI	*P*	HR	95% CI	*P*	HR	95% CI	*P*
	All-cause mortality								
Model 1*									
Q2	1.80	1.159–2.796	0.009	0.56	0.189–1.736	0.321	2.09	1.292–3.412	0.003
Q3	2.59	1.720–3.927	<0.001	0.98	0.396–2.472	0.982	2.80	1.766–4.455	<0.001
Q4	5.01	3.361–7.478	<0.001	1.64	0.680–3.982	0.269	4.97	3.166–7.820	<0.001
Model 2†									
Q2	1.44	0.928–2.241	0.104	0.53	0.173–1.622	0.266	1.62	0.997–2.639	0.051
Q3	1.82	1.202–2.759	0.005	0.83	0.332–2.077	0.691	1.87	1.176–2.990	0.008
Q4	3.12	2.087–4.688	<0.001	1.29	0.534–3.146	0.566	2.96	1.872–4.685	<0.001
Model 3‡									
Q2	1.87	1.166–3.014	0.010	0.59	1.173–2.028	0.404	2.19	1.299–3.709	0.003
Q3	2.40	1.530–3.773	<0.001	1.05	0.380–2.904	0.924	2.60	1.570–4.332	<0.001
Q4	3.67	2.360–5.719	<0.001	1.49	0.553–4.017	0.430	3.72	2.254–6.139	<0.001

CKD was defined as estimated GFR < 60 ml/min/1.73m^2^. Reference was Quartile 1 in model 1, 2, 3.

CKD = chronic kidney disease, HR = hazard ratio.

* Model 1: crude.

† Model 2: adjusted for age over 65, gender.

‡ Model 3: adjusted for age over 65, gender, serum albumin, urine albumin creatinine ratio, smoking status, body mass index, concurrent history of hypertension and diabetes mellitus.

## 4. Discussion

This study analyzed the association between serum Hcy concentration and all-mortality in CKD and nonCKD patients, using NHANES data. An increased mortality rate was observed in patients with high levels of Hcy in the nonCKD group. Moreover, we confirmed a significant correlation between the Hcy level and mortality risk; this risk was found to have a similar pattern after adjusting for confounding factors including age, gender, and inflammation or nutrition in the nonCKD group. However, in the CKD group, even in cases where the concentration of homocysteine was high, no association to mortality was observed. Therefore, this is consistent with the reverse epidemiology hypothesis of Hcy and mortality in CKD patients.^[6]^

Although it is well known that patients with CKD have a high CVD risk,^[15]^ there is little research on the mechanism through which this risk increases. Therefore, studying and identifying contributing factors other than these accompanying diseases are an important part of improving the prognosis of CKD patients. There are several potential traditional mechanisms, which can convert CKD to CVD. For example, systemic inflammation might lead to uncontrollable levels of uremic metabolite, such as phosphorus, that changes cardiac remodeling.^[4]^ Further, vascular calcification related to mineral bone disease in CKD patients (medial calcification) and inflammation such as C-reactive protein (CRP) or interleukin and endothelial dysfunction and metabolic disorder associated with adiponectin or FGF-23 are consider early biomarkers and etiologies causing excessive CVD occurrence of CKD.^[16,17]^ Prior studies have also considered Hcy as a risk factor, for CVD and cerebrovascular disease in the general population.^[5]^ While there have been cumulated hypotheses on the association between CVD occurrence and HHcy, Hcy is known to activate the associated pathway of atherogenesis or thrombosis by endothelial dysfunction, inflammation, and oxidative stress.^[18]^ In addition, studies have reported an association between patients with gene variance and metabolism in Hcy, such as methylenetetrahydrofolate reductase (MTHFR) 677C raise CVD risk with elevated Hcy. Finally, there have also been reports that the administration of folic acid or vitamin B12 can lower the Hcy level and reduce mortality.^[16,19]^ Owing to this evidence, HHcy has been recognized as a risk factor for CVD occurrence. The high prevalence of HHcy in patients with CKD has generated interest in the potential role of total homocysteine (tHcy) as a risk factor for the excess risk of CVD that is evident in this population.^[20–22]^

In 1969, Macully first announced in a postmortem case report that HHcy may be associated with atherosclerosis, which led to the concept of the “Hcy hypothesis,” that considered Hcy a potential risk factor for CVD, contributing to vascular physiology.^[23]^ Since then, observational studies have confirmed the association between HHcy and CVD. In the general population, HHcy is associated with arteriosclerosis, increased CVD risk, and mortality. In addition, even in patients with coronary artery disease, it is associated with poor prognosis and is considered a prognostic factor of poor outcome in patients with Type 2 DM.^[24]^ However, there is a lack of research on the risk perceptions of CVD in CKD patients. Hcy indicates of vascular disease, especially in Asian populations reporting insufficient daily folic acid intake, compared to their Western counterparts.^[25]^ Since folate and vitamin B12 act as cofactors in Hcy metabolism, insufficiency of these cofactors could cause HHcy.^[26]^ Therefore, there have been several studies on the improvement in CVD outcome after administration of vitamin B12 and folate for the treatment of HHcy. In contrast, a systematic review and meta-analyses revealed that Hcy-lowering treatment did not improve CVD outcomes in patients with a high level of Hcy among the ESRD population.^[12]^ Prior studies considered an increase in Hcy concentration not being the cause of CKD, but the consequence of CKD, caused by the decrease in glomerular filtration rate (GFR).^[27,28]^ However, recent studies suggest that HHcy may be a risk factor of CKD itself. The results of a meta-analysis of 41 studies showed that, higher Hcy was associated with a higher GFR decline over time.^[29]^ Studies on elderly patients with hypertension in China reported that HHcy can predict a decline of kidney function.^[30]^ Furthermore, CVD may also occur after CKD through various mechanisms such as increased Hcy concentration, oxidative stress, and endothelial dysfunction.^[31]^ However, results on the effect of HHcy on death in advanced CKD patients, are inconclusive.

Although BMI, serum cholesterol, and high blood pressure are traditional risk factors for cardiovascular disease and mortality in the general population, in patients with CKD with regards to Hcy, the results of prior studies reveals the effect of reverse epidemiology in these traditional risk factors.^[32]^ Although Hcy is known to be increased in CKD patients,^[33,34]^ findings regarding whether it is an independent risk factor for increasing CVD risk in CKD patients are inconclusive.^[35–38]^ In 2 studies with 367 and 88 hemodialysis patients, respectively, the low Hcy level in ESRD patients was observed to increase CVD outcome; moreover, Hcy also showed a reverse epidemiology pattern as mentioned above.^[39,40]^ This study also revealed at the correlation between Hcy levels and cardiovascular mortality in participants with and without CKD (Supplementary Tables 2 and 3, http://links.lww.com/MD/H159). In the absence of propensity score matching, the findings remained similar to all-cause mortality. However, in the analysis conducted after propensity score matching, no group among the CKD or nonCKD category, showed statistically significant outcomes with Hcy level. As a result of the reduction in the size of the sample after matching the propensity score, this can be regarded favorable to the main hypothesis. In spite of this, the findings of our research indicate that HHcy has a significant correlation with mortality rates in the general population. What more, the impact remained stable even after a number of potential confounding variables were accounted for. On the other hand, among patients with CKD, the level of Hcy was not observed to be linked with mortality before or after the use of propensity matching, which indicates a pattern that corresponds to reverse epidemiology.

As mentioned above, CKD is particularly known as a representative disease, causing reverse epidemiology. Many researchers explain this inverse correlation using several mechanisms. First, inflammation and protein energy wasting (PEW), which occur in advanced CKD patients, have been discussed. Previous studies showed that tHcy decreased when CKD patients faced inflammation or PEW.^[40–42]^ In addition, prior studies report an association between inflammations, PEW, and poor CVDs.^[43,44]^ Similarly, the study observed a reduction in albumin and Hcy level in advanced CKD patients. Therefore, PEW can offset the effect of HHcy on the clinical outcome including mobility. The second possible explanation is selection bias, which included survival bias. This is also a pattern that is observed in epidemiology studies among elderly and advanced CKD patients, including ESRD patients.^[45]^ As these patients have high morbidity and mortality, compared to that of the general population, they may be viable targets for cross-section studies. In addition, even among CKD patients, there is an immense difference in epidemiological characteristics between patients who have just begun dialysis and those who have elapsed time.^[46]^ Finally, in describing reverse epidemiology, time discrepancy is a competitive risk factor that can affect mortality. This can be explained first with the obesity paradox, as overweight or obesity is known to be highly related to CVD outcomes in developed countries such as the United States and Europe with high average life expectancy.^[47,48]^ Nevertheless, in developing countries, under-nutrition is known to be a powerful factor that can predict a poor clinical outcome, including mortality.^[49]^ Therefore, for CKD patients with low life expectancy, strict management of weight, blood pressure, DM, and low serum cholesterol and Hcy maintenance, which are known to be highly associated with long-term survival, maybe less important to improve clinical outcome, compared with the general population. Thus, the presence of reverse epidemiology may not necessarily imply that the principles of vascular pathophysiology are different in CKD patients. However, it might indicate that other superimposed factors, such as PEW and inflammation, are more important.

As a limitation of this study, only eGFR served as the basis of the study definition of CKD. A total of 10,021 patients were investigated, of which, 126 were excluded from the research due to their insufficient eGFR determined by Creatinine. Ultimately, 9895 participants were included in the investigation, out of which, 160 had a UACR deficiency. This included patients with A1 albuminuria (N = 8605), A2 (N = 946), and A3 (N = 184) (Supplemental Digital Content (Table S1, http://links.lww.com/MD/H160)). In contrast, very few A3 patients were included in the analysis of dietary data for health surveys. This factor strengthened the decision to follow the eGFR grade. Since fewer patients matched the A3 criteria, chronic kidney disease was classified as an eGFR of <60 mL/min/1.73 m^2^.

## 5. Conclusion

This study did not find a correlation between the Hcy level and mortality rate among the CKD group, unlike among the nonCKD group. This altered pattern of risk factors, can be attributed to the wastage of protein-energy, or the chronic inflammation status accompanied by CKD.

## Author contributions

Research idea and study design: KDY, GSK; data acquisition: JHS, HH, KDY, GSK; data analysis/interpretation: HH, JHS, GSK, KDY; supervision or mentorship: EB, JL, JPL, JSL. Each author contributed important intellectual content during manuscript drafting or revision. All authors read and approved the final manuscript.

## Supplementary Material


