# Supplementary material for: Association between homocysteinemia and mortality in CKD: A propensity-score matched analysis using NHANES-National Death Index
Source: Medicine (Baltimore). 2022 Sep 9;101(36):e30334. doi: 10.1097/MD.0000000000030334 (PMC10980502; doi:10.1097/MD.0000000000030334)

Supplemental Digital Content (Figure S1). Standardized difference (SD) before and after propensity-score matching

(A) Change of SD value for before and after propensity-score matching by variables

(B) Distribution of SD for before and after propensity-score matching

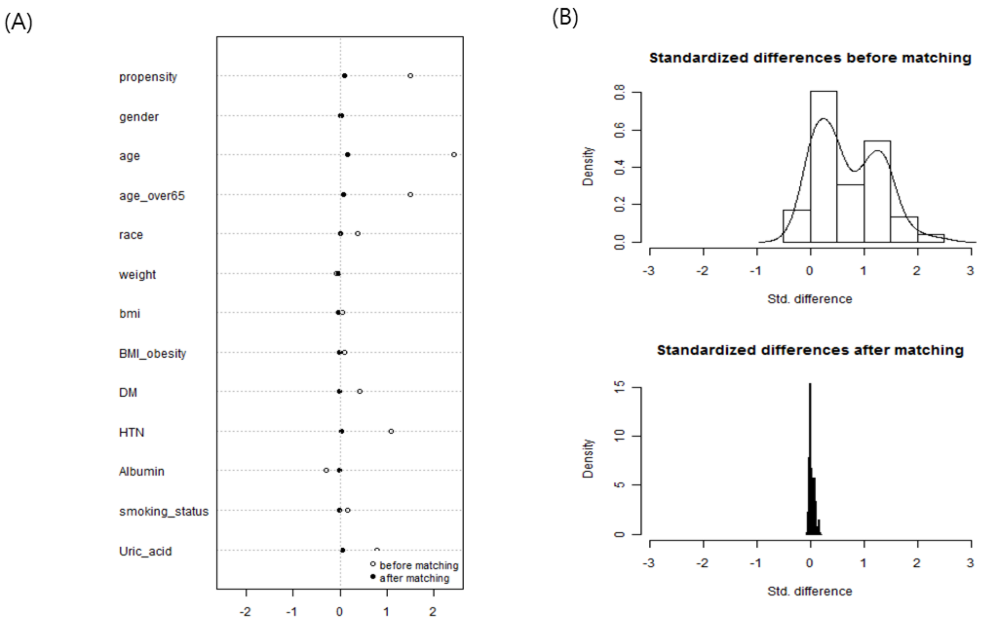

Supplement: Supplementary file 1 [file medi-101-e30334-s001.pdf]
