# Supplementary material for: Association between homocysteinemia and mortality in CKD: A propensity-score matched analysis using NHANES-National Death Index
Source: Medicine (Baltimore). 2022 Sep 9;101(36):e30334. doi: 10.1097/MD.0000000000030334 (PMC10980502; doi:10.1097/MD.0000000000030334)
Supplement: Supplementary file 2 [file medi-101-e30334-s002.pdf]

Table S2. Hazard ratios of cardiovascular mortality according to homocysteine level (quartile)

	All (n=9895)			CKD (n=1025)			Non-CKD (n=8870)		
	HR	95% CI	*P*	HR	95% CI	*P*	HR	95% CI	*P*
	Cardiovascular mortality								
Model 1†									
Q2	1.86	1.108-3.123	<0.001	0.46	0.103-2.062	0.311	1.95	1.125-3.403	0.017
Q3	3.20	1.985-5.183	<0.001	0.44	0.132-1.532	0.201	1.74	1.749-5.002	<0.001
Q4	13.61	8.806-21.052	<0.001	0.97	0.301-3.057	0.963	6.10	6.109-16.102	<0.001
Model 2‡									
Q2	1.21	0.717-2.047	0.473	0.42	0.095-1.908	0.265	1.20	0.688-2.118	0.511
Q3	1.43	0.873-2.364	0.154	0.36	0.107-1.257	0.111	1.28	0.742-2.224	0.372
Q4	3.82	0.577-0.878	<0.001	0.74	0.235-2.340	0.609	3.06	1.809-5.175	<0.001
Model 3§									
Q2	1.21	0.705-2.092	0.485	0.88	0.280-2.775	0.830	1.70	1.301-2.240	<0.001
Q3	1.39	0.829-2.340	0.211	1.23	0.449-3.373	0.687	1.99	1.523-2.604	<0.001
Q4	3.08	1.879-5.054	<0.001	1.93	0.720-5.182	0.191	3.01	2.308-3.941	<0.001

CKD was defined as estimated GFR < 60 ml/min/1.73m^2. Reference was Quartile 1 in model 1, 2, 3

† Model 1: crude, ‡Model 2: adjusted for age over 65, gender, §Model 3: adjusted for age over 65, gender, serum albumin, urine albumin creatinine ratio, smoking status, body mass index, concurrent history of hypertension and diabetes mellitus

CKD = chronic kidney disease, HR = hazard ratio

Table S3. Hazard ratios of cardiovascular mortality according to homocysteine level (quartile) after propensity score matching

	All (n=2756)			CKD (n=741)			Non-CKD (n=2015)		
	HR	95% CI	*P*	HR	95% CI	*P*	HR	95% CI	*P*
	Cardiovascular mortality								
Model 1†									
Q2	0.89	0.433-1.864	0.774	0.24	0.040-1.437	0.118	1.13	0.497-2.592	0.764
Q3	1.28	0.666-2.458	0.459	0.42	0.112-1.479	0.179	1.43	0.666-3.084	0.357
Q4	2.94	1.600-5.428	2.947	0.669	0.211-2.123	0.496	3.44	1.671-7.115	0.001
Model 2‡									
Q2	0.70	0.340-1.469	0.353	0.23	0.039-1.415	0.114	0.86	0.378-1.981	0.731
Q3	0.84	0.436-1.629	0.610	0.34	0.098-1.197	0.093	0.91	0.414-1.958	0.792
Q4	1.69	0.910-3.166	0.096	0.48	0.152-1.555	0.224	1.89	0.904-3.979	0.091
Model 3§									
Q2	0.77	0.362-1.666	0.516	0.12	0.013-1.251	0.077	1.03	0.431-2.473	0.943
Q3	0.96	0.483-1.916	0.912	0.35	0.102-1.251	0.107	1.09	0.479-2.501	0.830
Q4	1.68	0.873-3.247	0.120	0.47	0.147-1.536	0.214	2.00	0.903-4.444	0.087

CKD was defined as estimated GFR < 60 ml/min/1.73m^2. Reference was Quartile 1 in model 1, 2, 3

† Model 1: crude, ‡Model 2: adjusted for age over 65, gender, §Model 3: adjusted for age over 65, gender, serum albumin, urine albumin creatinine ratio, smoking status, body mass index, concurrent history of hypertension and diabetes mellitus

CKD = chronic kidney disease, HR = hazard ratio
