# Supplementary material for: Association between homocysteinemia and mortality in CKD: A propensity-score matched analysis using NHANES-National Death Index
Source: Medicine (Baltimore). 2022 Sep 9;101(36):e30334. doi: 10.1097/MD.0000000000030334 (PMC10980502; doi:10.1097/MD.0000000000030334)
Supplement: Supplementary file 3 [file medi-101-e30334-s003.pdf]

Table S1. CKD by gradation of color according to KDIGO classification in the study population[1].

N=9895	Yellow	Orange	Red	Total
**N**	1237	342	219	1798
**Percentage(%)**	12.5	3.45	2.21	18.17

REFERENCE

1. Levey AS, de Jong PE, Coresh J, El Nahas M, Astor BC, Matsushita K, et al. The definition, classification, and prognosis of chronic kidney disease: a KDIGO Controversies Conference report. Kidney Int. 2011;80(1):17-28. doi: 10.1038/ki.2010.483. PubMed PMID: 21150873.
